# RESCUE- expected usefulness and willingness to participate in a trauma-informed group intervention for coping with traumatic work experiences in the emergency medical services

**DOI:** 10.3389/fpsyt.2026.1846003

**Published:** 2026-05-12

**Authors:** Jana Austel, Sabine Schmitt, Alexander Behnke, Jacqueline Lehmann, Inga Schalinski

**Affiliations:** 1 Department of Human Sciences, University of the Bundeswehr Munich, Neubiberg, Germany; 2 Non-Governmental Organization vivo International e.V., Konstanz, Germany; 3 Department of Clinical and Biological Psychology, Institute for Psychology and Education, Ulm University, Ulm, Germany; 4 German Center for Mental Health, Das Deutsche Zentrum für Psychische Gesundheit (DZPG), Partner Site Mannheim-Heidelberg-Ulm, Ulm, Germany; 5 The Bavarian Science Alliance for Peace, Conflict and Security Research, Munich, Germany

**Keywords:** burnout symptoms, critical incident-related stress, emergency medical services, facts derived from narrative exposure therapy (NETfacts), group-based treatment

## Abstract

Emergency Medical Services personnel (EMS) are confronted with potentially highly stressful and traumatic occupational experiences, placing them at high-risk for mental disorders. Avoidant coping mechanisms, internalized and occupational stigma not only impede disclosure of and processing the incident-related stress but also weaken the overall resilience of the EMS. Effective interventions are needed that operate both at the individual and group level. The aim of this study was to investigate the expected usefulness and willingness to participate in a trauma-informed intervention (Facts derived from Narrative Exposure Therapy, NETfacts). A total of 256 German EMS (67.19% men, 32.81% women) participated in the online survey. We assessed the expected usefulness and willingness to participate in NETfacts, critical incident-related stress (EMS Critical Incident Inventory EMS-CII), burnout symptoms (Professional Quality of Life ProQOL), age and work experience. Overall, about half of the EMS expected NETfacts to be at least somewhat useful, and reported a generally willingness to participate. Expected usefulness was neither associated with critical incident-related stress nor burnout symptoms. Younger participants (<35 years) showed a generally higher willingness to participate than older participants. However, the willingness is across both age groups positively associated with higher levels of critical incident-related stress. Nevertheless, burnout symptoms and the willingness are negatively associated among participants age 35 and older, while remaining stable among their younger colleagues. Early, trauma-informed and age-sensitive prevention programs are needed to mitigate the adverse effects of critical incidents among EMS. Our study presents EMS preferred circumstances to enhance employees’ uptake of such a program.

## 1 Introduction

Emergency Medical Services personnel (EMS, e.g., paramedics, emergency doctors) operate in highly demanding work environments, which is associated with increased risk of for mental illness (1–3). Despite the urgent need for interventions, rates of mental disorders remain higher than in the general population (4) and employees’ uptake of programs is limited (5). The present study aimed to examine EMS preferred program characteristics, expected usefulness and willingness to participate in a novel trauma-informed group intervention, potential influential factors such as critical incident-related stress, burnout symptoms, age and work experience, with the goal of informing recruitment and implementation.

Two core aspects of the highly demanding work environments include challenging organizational structures - such as long and irregular working hours, shift work and physically demanding tasks (6–8) - and exposure to multiple potentially highly stressful and traumatic incidents (9, 10). Pediatric events and severe injuries have been reported as two particularly stressful incidents (9, 11). These critical incidents can accumulate and increase the risk of mental disorders (building block effect) (7), including burnout (3, 12), affective disorders (1), anxiety disorders (2) and posttraumatic stress disorder (4, 13). While Palfi et al. reported higher vulnerability among younger EMS (3), other studies have shown age as non-significant predictor for mental disorders (14, 15).

How EMS cope with these critical incidents plays a crucial role for the experienced incident-related stress (16). Common coping strategies include acceptance, problem solving, reappraisal, and avoidance (17–20). Avoiding the memory of a critical incident can thereby be understood as the human need to block out aversive and focus on positive experiences, and has shown associations with increased psychological well-being (20), indicating a buffering function (21). However, if avoidant coping is used continuously, it can become maladaptive (22) and ultimately contribute to the maintenance or even elevation of stress, burnout or posttraumatic stress disorder at the individual level (18, 19). At the group level, avoiding to talk about incident-related stress can function as barrier to receive peer support. Research has shown that social support has beneficial effects for mental health (23) and is perceived also among EMS as useful to cope with critical incidents (17, 24), whereas insufficient social support and feelings of loneliness have been associated with an increased risk for depression and posttraumatic stress disorder (25–28).

However, to receive social support, some degree of disclosing one’s incident-related stress is required. In a study of Depierro et al. (29), one in five World Trade Center respondents stated that they preferred to be able to cope on their own, indicating a limited openness to disclose their incident-related stress to others. While research has shown no influential effects of age or attitudes toward mental health support on disclosure (30), disclosure appears to decrease with greater work experience (31). Potential barriers include concerns about career-related consequences, fear of stigmatization (28, 32) and experienced stigmatization (5, 28, 30, 33). Moreover, EMS were shown to have negative prejudices towards mental It is likely that disclosure is also avoided due to internalized stigma, based on research showing prejudices towards mentally ill people among EMS (34). In a policy paper, Butollo et al. (35) emphasized the importance of becoming aware of the issue and the value of disclosure, which is further supported by research suggesting that when EMS disclose their incident-related stress, this can further promote help-seeking behaviors in peers and strengthen overall peer support (28).

First evidence from qualitative studies indicates that training peers to support colleagues in their incident-related stress can reduce stigma and increase help-seeking behavior (36) and that combining psychoeducation, peer-support and, if needed, trauma exposure by a mental health professional (using Eye Movement Desensitization and Reprocessing) may have beneficial effects on psychological well-being, feeling less alone and being more willing to talk about own incident-related stress (37). The majority of prevention programs includes interventions about stress management and resilience (38) and shows limited effectiveness (39). Facts derived from Narrative Exposure Therapy (NETfacts) is a trauma-informed intervention that aims to decrease (internalized) stigmatization and to increase peer empathy and support as well as survivors’ willingness to seek social or professional help if needed (40). NETfacts combines group sessions – addressing survivor’s ‘typical’ peritraumatic experience and the importance of posttraumatic social reactions - with a singular individual exposure session using Narrative Exposure Therapy (41) by a mental health professional, if needed. First results show, among others, reduced stigmatization toward sexual violence survivors and ex-combatants as well as increased help-seeking behavior within traumatized communities in the Eastern Democratic Republic of the Congo (42), as well as decreased internalized stigma among survivors of childhood maltreatment in Germany (43).

Our primary goal of this study was to adapt and pilot NETfacts for EMS in Germany. However, due to unsuccessful recruitment despite extensive efforts, we conducted an online survey to explore the factors underlying expected usefulness, willingness to participate and preferred characteristics of NETfacts under which participation might be more likely, resulting in the following research questions: 1) Which circumstances (e.g., group size, preferred familiarity among the participants, number of sessions, offline or online setting) are preferred for participating in NETfacts? Is this intervention expected to be useful and are participants willing to participate? Upon replication of the relationship between critical incident-related stress and burnout symptoms as well as the examination of age and work experience as potential moderators of this relationship, the following questions were investigated: 2) Are critical incident-related stress and burnout symptoms associated with (a) the expected usefulness of NETfacts and (b) the willingness to participate, and are these associations moderated by age and work experience?

## 2 Materials and methods

### 2.1 Study design and procedure

This cross-sectional online study was approved by the ethical commission of the University of the Bundeswehr (EK UniBw M 25–19 Participants were recruited via multiple channels, combining convenience and network-based methods. Personal contacts were established by Jacqueline Lehmann and Jana Austel, both authors of this manuscript, who have a professional background in EMS. In addition, study information was disseminated via social media through professional networks and contacts established at EMS-related conferences, where colleagues were asked to share the study flyer within their networks. Furthermore, invitation letters were sent by the research team on behalf of [institution name] to 331 ambulance stations and fire departments across Germany. These letters included a brief description of the study objectives, eligibility criteria, and information about voluntary and anonymous participation. The study was implemented via the platform SoSci Survey (44), with participation open from May 2025 to March 2026. Inclusion criteria was EMS affiliation, regardless of their qualifications or kind of employment (e.g., full-time or part-time). Participation was voluntary and without incentive. All participants provided digital informed consent before participation.

### 2.2 Sample characteristics

A total of 260 EMS participated in the survey. Following the recommendations of Song and Shepperd (45), four participants were excluded because of more than 40% missing data in incomplete questionnaires, resulting in a final sample size of *N* = 256, with 172 men (67.2%) and 84 women (32.81%), 131 (51.17%) worked as ‘Notfallsanitäter’, which is comparable to paramedics, 78 (30.47%) as ‘Rettungssanitäter’, which is comparable to emergency medical technician, 14 (5.47%) as an emergency doctor and 32 (12.50%) with other qualifications (e.g., trainees). The majority (*n =* 184, 72%) worked on a rotating shift schedule, including alternating day and night shifts. Sample characteristics are displayed in **Table 1**.

**Table 1:** Sample characteristics.

Qualitative Question	Answers	n	%
Demographics			
Gender^*1^	Male	172	67.19%
	Female	84	32.81%
Age	Younger than 35	151	58.98%
	Aged 35 years and older	105	41.02%
Occupational structures			
Organization ^*2^ (Multiple responses were allowed)	EMS provider (e.g., German red cross)	246	96.09%
	EMS within fire departments	46	17.97%
Role	Paramedic	140	54.69%
	Emergency medical technican	78	30.47%
	Emergency doctor	14	5.47%
	Others (e.g. trainee, water rescue)	24	9.37%
Work experience in years	Five years and less	88	34.38%
	More than five years	168	65.62%
Field of operation	Urban	91	32.55%
	Rural	62	24.22%
	Mixed	103	40.23%
Shift schedule	Rotating	184	71.88%
	Mainly day shifts	28	10.94%
	Mainly night shifts	9	3.52%
	Others (e.g., 24 hours shifts)	35	13.67%
Access to preventive interventions			
Does your organization offer any interventions to aid coping with critical incident-related stress?	Yes	197	76.95%
	No	21	8.20%
	I don’t know	37	14.45%
	I can’t/don’t want to answer	1	0.39%
Of those with organizational offers:			
Did you participate in one of the interventions or would you do so if needed?	Yes	96	48.73%
	No	99	50.25%
	I don’t want to answer	2	1.02%
Is the provided intervention sufficiently useful?	Yes	80	40.61%
	No	48	24.37%
	I don’t know	69	35.03%

*N =* 256; ^*1^ None reported non-binary or other gender identities; ^*2^ multiple responses were allowed, therefore, percentages can exceed 100%.

### 2.3 Measures

*Critical events at work*. Exposure to critical incidents was measured using the EMS Critical Incident Inventory (EMS-CII) (9, 46). The EMS-CII assesses 33 critical incidents in the context of emergency medical service missions, covering direct traumatization (e.g., incident involving a self-inflicted traffic accident during emergency response, resulting in injury to another person), indirect traumatization (e.g., incident involving many (>5) injured or dead) and exposure to aversive detail of traumatic events at work (e.g., incident involving exposure to human body parts) and further allows participants to report another highly aversive/stressful event as free text. Participants reported incident experience (0 = not experienced, 1 = experienced). For each incident type experienced, participants were instructed to remember the most stressful event of this type and to rate which impact of this event has on them until the present day on a six-point Likert scale (0 = not at all, 5 = extremely burdening). The cumulative impact of critical incidents was calculated as the sum of impact-of-event ratings across experienced incident types. The internal consistency of the cumulative impact of events was excellent with Cronbach’s α = .91.

*Burnout symptoms*. Burnout symptoms were operationalized as compassion fatigue with feelings of helplessness and difficulties in daily work life and measured with the 10-item Professional Quality of Life (ProQOL, 46, 47, 47), using a five point Likert scale (1 = never, 5 = very often/always). A sum score was calculated representing the severity of compassion fatigue. Internal consistency was acceptable with Cronbach’s α = .73.

*Attitudes towards NETfacts.* Additionally, the intervention NETfacts was introduced as follows: ‘EMS experiences potentially stressful events in their working life which can cause psychological burdens. Compared to civilians, EMS have higher prevalences of mental illness. Furthermore, EMS often don’t speak about their experiences because they fear stigmatization or criticize themself. As we can see in other populations, the common exchange in a group can help reflecting these rescue events and can lead to more wellbeing, more group-coherence and higher psychological resilience. In the new group-based intervention some exemplary rescue events were read aloud and reflected by the group. Moreover, based on personal experiences influences of the working environment will be discussed and reflected how they can be supportive. Voluntarily the participants can reflect a personal stressful rescue event in a one-on-one session with a psychotherapist.’ Participants were asked to indicate on a five-point Likert scale the likelihood of the intervention’s usefulness (1 = very unlikely, 5 = very likely), their willingness to participate (1 = not at all willing, 5 = very willing), and their preferred circumstances for implementation (e.g., group size, familiarity among participants, number of sessions, see **Table 2**). These questions were developed by the study team.

**Table 2:** Descriptive statistics: expected usefulness, willingness to participate and group preferences.

Qualitative question	Preferences	n	%
How likely do you think it is that the described group approach would be useful for you and your professional environment?	Very unlikely	12	4.69%
	Unlikely	45	17.58%
	Undecided	67	26.17%
	Likely	95	37.11%
	Very likely	37	14.45%
If the circumstances of the group intervention align with your preferences, how willing would you be to participate?	Not at all willing	9	3.52%
	Rather not willing	20	7.81%
	Perhaps willing	59	23.05%
	Rather willing	103	40.23%
	Very willing	65	25.39%
Which group size do you prefer?	Up to 7 persons	193	75.39%
	Between 8–15 persons	37	14.45%
	Between 16–25 persons	2	0.78%
	More than 25 persons	1	0.39%
	I dont know	23	8.98%
Which familiarity among the participants do you prefer?	Only EMS from my team	66	25.78%
	Mixed group of familiar EMS	138	53.91%
	Only unfamiliar EMS	52	20.31%
Preferred number of sessions (with 1, 5 to 2 hours per session)	No session	46	17.97%
	One session	64	25.00%
	Two sessions	86	33.59%
	Three sessions	31	12.11%
	Four sessions	29	11.33%
At what times is a personal participation possible for you? ^*^	Morning (9 to 11 a.m.)	105	41.02%
	Early afternoon (1 to 3 p.m.)	67	26.17%
	Later afternoon (3 to 5 p.m.)	80	31.25%
	Evening (6 to 8 p.m.)	107	41.80%
	Weekend	47	18.36%
	No possibility to participate	20	7.81%
Can you imagine reflecting on a rescue event in a one-on-one session with a psychotherapist?	Yes	186	72.66%
	No	19	7.42%
	Maybe	51	19.92%
Is an online format useful?	Not useful	100	39.06%
	Somewhat useful	62	24.22%
	Useful	94	36.72%

*N =* 256, ^*^ multiple responses were allowed.

### 2.4 Statistical analyses

All calculations were conducted in R 4.4.1 (48) with a significance level of α = 5%. There was one missing value for one participant on one item of the EMS-CII which was imputed using multiple imputation (49). The first research question was addressed using descriptive statistics. Spearman rank-correlations were computed to examine bivariate associations. Although the dependent variables were ordinal, ordinary least square regression according to Hayes (50) was used as a robust and widely applied approximation for Likert-type outcomes. This choice also facilitates comparability with established regression-based moderation framework. To ensure the stability of model estimates moderation analyses were conducted using 10000-times bootstrapping (non-parametric bootstrapping). Prior to moderation analyses, all continuous predictor variables were mean-centered to reduce multicollinearity. Because the dependent variables were ordinal, cumulative link models were additionally applied for sensitivity analyses. Due to the low sample size and the high correlation between age (coded as 0 < 35 years and 1 ≥ 35 years) and work experience (coded as 0 for ≤ 5 years and 1 for > 5 years) (*r_ф_ = .*47, *p* <.001, 95% CI [.37,.56]), separate moderation analyses including age and work experience were conducted.

## 3 Results

### 3.1 Aim 1 preferred group characteristics

More than half of participants estimated NETfacts to be at least ‘likely useful’ (*n* = 132, 51.56%), and the majority reported to be at least ‘perhaps willing’ to participate (*n* = 227, 88.67%), if the circumstances align with their preferences. Preferences included two face-to-face sessions (*n* = 86, 33.59%), either held in the morning (*n* = 105, 41.02%) or in the evening (*n* = 107, 41.80%) with groups up to seven persons (*n* = 193, 75.39%), mixed of familiar and unfamiliar EMS (*n* = 138, 53.91%). Descriptive results are displayed in **Table 2**.

### 3.2 Replication of the association between critical incident-related stress and burnout symptoms

Participants reported about 20 critical incident types (*Mdn =* 20.5, *Q1 – Q3 = *16.0 - 24.0) in the context of EMS missions. The most frequently reported incidents involved self- and other-endangerment (*n =* 246, 96%), failed resuscitation (*n* = 243, 95%), or patients facing social emergencies (*n* = 242, 95%). Particularly critical incidents with enduring incident-related stress were potentially life-threatening pediatric emergencies (*Mdn* = 1, *Q1- Q3 = *0.00 - 3.00) and incidents in which the actual situation involved substantially more severe suffering or danger than initially reported (*Mdn =* 1, *Q1 – Q3 = *0 - 2.00). Overall critical incident-related stress was *Mdn* = 21 (*Q1 – Q3* = 10.00 - 31.00). Critical incident-related stress was not correlated with age, but showed a significant positive correlation with work experience and burnout symptoms (**Supplementary Table 1**). Age showed no main effect (*b* = -0.18, 95% CI [-1.40, 1.10], *p = .*772), or moderation effect on the relationship between critical incident-related stress and burnout symptoms (*b* = 0.00, 95% CI [-0.07, 0.08], *p = .*899). Similarly, work experience showed no main effect (*b* = -0.59, 95% CI [-1.86, 0.69], *p = .*371), or moderation effect (*b* = 0.05, 95% CI [-0.02, 0.13], *p = .*180).

### 3.3 Aim 2: expected usefulness of NETfacts

Moderation analyses did not reveal significant main effects of either critical incident-related stress or burnout symptoms on the expected usefulness of NETfacts (**Table 3**, **Supplementary Table 2**). Moreover, neither age (**Table 3**) nor working experience (**Supplementary Table 2**) indicate any main or moderation effects of these variables. Additionally, Cumulative Link Models revealed no significant effects associated with age (**Supplementary Table 3**) or work experience (**Supplementary Table 4**).

**Table 3:** Regression analyses including age as a moderator.

Outcome and model fit	Predictors	*b (SE)*	95%CI (Boot)	*β*	*p*
Outcome: Expected usefulness Model fit: *F*(3, 252) = 1.46, *p* = .227, *R^2^_adj_* = .01	Constant	3.47 (0.08)	[3.31, 3.63]		<.001
	Critical incident-related stress (EMS-CII)	0.00 (0.01)	[-0.01, 0.01]	.07	.409
	Age (coded as 1 for ≥35 years and 0 for <35 years)	-0.20 (0.14)	[-0.48, 0.06]	-.09	.139
	Moderation (age x critical incident-related stress)	0.00 (0.01)	[-0.01, 0.02]	.04	.669
Outcome: Expected usefulness Model fit: *F*(3, 252) = 1.89, *p* = .132, *R^2^_adj_* = .01	Constant	3.46 (0.09)	[3.30, 3.62]		<.001
	Burnout symptoms (ProQOL)	-0.02 (0.02)	[-0.05, 0.02]	-.07	.397
	Age (coded as 1 for ≥35 years and 0 for <35 years)	-0.18 (0.14)	[-0.44, 0.10]	-.08	.201
	Moderation (age x burnout symptoms)	-0.02 (0.03)	[-0.07, 0.04]	-.06	.475
Outcome: Willingness to participate Model fit: *F*(3, 252) = 6.32, *p* <.001, *R^2^_adj_* = .06	Constant	3.89 (0.08)	[3.75, 4.04]		<.001
	Critical incident-related stress (EMS-CII)	0.01 (0.00)	[0.01, 0.02]	.26	<.001
	Age (coded as 1 for ≥35 years and 0 for <35 years)	-0.31 (0.13)	[-0.57, -0.06]	-.15	.015
	Moderation (age x critical incident-related stress)	-0.00 (0.01)	[-0.02, 0.01]	-.03	.689
Outcome: Willingness to participate Model fit: *F*(3, 252) = 3.21, *p* <.005, *R^2^_adj_* = .03	Constant	3.87 (0.08)	[3.71, 4.02]		<.001
	Burnout symptoms (ProQOL)	0.01 (0.02)	[-0.02, 0.03]	.04	.677
	Age (coded as 1 for ≥35 years and 0 for <35 years)	-0.26 (0.14)	[-0.51, 0.00]	-.12	.049
	Moderation (age x burnout symptoms)	-0.05 (0.03)	[-0.09, 0.00]	-.17	.049

*N* = 256; 95%CI = bootstrap-based confidence interval, with 10000 times (non-parametric) bootstrap intervals; critical incident-related stress (EMS-CII = EMS Critical Incident Inventory) and burnout symptoms (ProQOL = Professional Quality of Life) were centered prior to calculation.

### 3.4 Aim 2: willingness to participate in NETfacts

**Figure 1:**
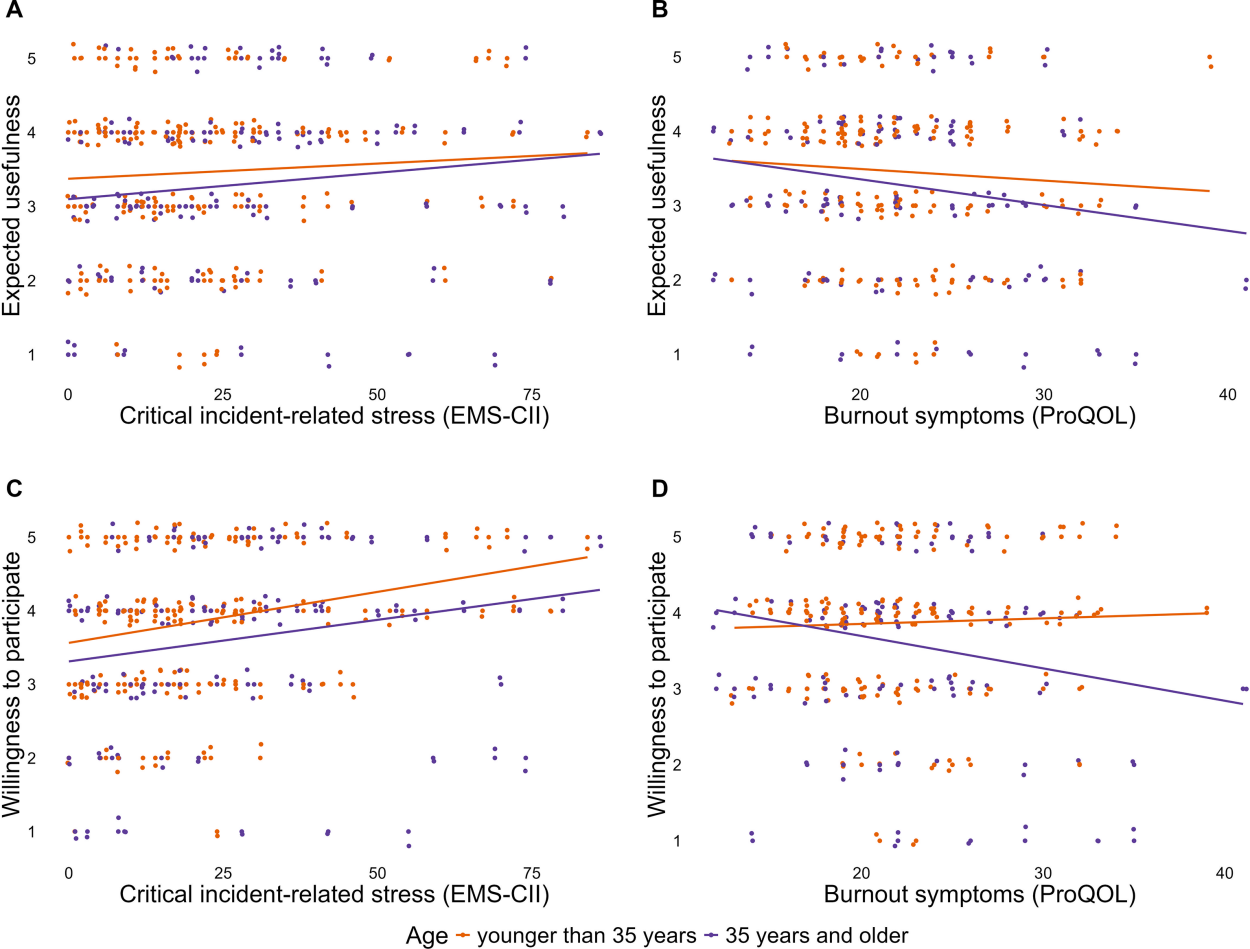
Expected usefulness **(A, B)** of NETfacts and willingness to participate **(C, D)** among EMS moderated by age (*N* = 256). Each dot represents one participant and is color-coded by age group; lines indicate linear simple slopes for each group. Orange denotes participants < 35 years, and purple denotes participants aged *≥* 35 years.

Critical incident-related stress had a significant positive main effect on willingness to participate (**Table 3**). Furthermore, age had a significant main effect, indicating that both younger than 35 year old and 35 years and older EMS are more willing to participate with increasing critical incident-related stress (**Table 3**, **Figure 1C**). Cumulative Link Models showed similar significant main effects of critical incident-related stress, whereas age did not show any significant effects (**Supplementary Table 3**). Moderation analysis revealed neither a significant main effect nor a significant moderation effect of work experience for willingness to participate (**Supplementary Table 2**). Cumulative Link Model corroborate these results (**Supplementary Table 4**).

In moderation analyses, burnout symptoms showed no significant main effect on participants’ willingness to participate in NETfacts (**Table 3**, **Supplementary Table 2**). However, this association is significantly moderated through age, indicating that participants aged 35 years and older are less willing to participate at higher levels of burnout symptoms (**Table 3**, **Figure 1D**). In the Cumulative Link Model, age did not significantly moderate this association (**Supplementary Table 3**). Additional moderation analysis showed no significant main or moderator effects for work experience (**Supplementary Table 2**). Similarly, the Cumulative Link Model revealed no significant effects including work experience (**Supplementary Table 4**).

## 4 Discussion

### 4.1 Summary

This study examined German EMS’ preferred characteristics, expected usefulness and willingness to participate in NETfacts, a trauma-informed group approach (40) - building on prior recommendations and qualitative research that emphasized disclosure, peer support and, if needed, trauma exposure treatment with a mental health professional to be of particular importance for EMS (35, 39). We further investigated the relationship between critical incident-related stress and burnout symptoms, as well as their associations with expected usefulness of NETfacts and willingness to participate, including age and work experience as moderators.

In line with prior research, our sample operates in highly demanding work environments with long and irregular working hours, shift work (6–8) and exposure to multiple occupational critical incidents (9, 10), placing them at high risk for mental disorders (1–3). While the majority of our sample reported that their aid organizations offer interventions to help cope with critical incident-related stress, about half of the participants stated to have not participated or would not participate if needed. Almost one third further stated that the existing interventions are not sufficiently useful. These hesitations are in line with prior research indicating that EMS’ uptake of existing programs is limited (5). Regarding our first aim, approximately half of the participants rated NETfacts as at least likely to be useful, and more than 65% indicated that they would be at least rather willing to participate, provided that the intervention characteristics align with their preferences. Most participants preferred a mixed group of familiar and unfamiliar EMS, with a group size up to seven. Additionally, there was a clear preference for a brief format of two group sessions, scheduled either in the morning or evening. Notably, over 70% expressed interest in processing a personal critical experience within an individual session with a mental health professional. Preferences regarding delivery mode were relatively balanced, with similar proportions favoring in-person and online formats.

In contrast to other studies (3, 12), the present sample appeared to exhibit comparatively lower levels of burnout symptoms (51). As expected, a significant positive correlation between critical incident-related stress and burnout symptoms has been confirmed (16). These results are in line with assumptions of the building block effect (52), indicating that cumulated stressful events can lead to mental burden. Age emerged not as a significant moderator of this relationship, which is congruent with other previous studies (14, 15), but appears to contradict the study by Palfi et al. (3). However, Palfi et al. (3) reported a significant effect of age solely for depersonalization. While depersonalization has shown associations with burnout symptoms among first responders (53), it is not measured by the ProQOL which has been used in our study.

No significant relationships were found between critical incident-related stress or burnout symptoms and the expected usefulness of NETfacts. In contrast, critical incident-related stress was significant positively associated with willingness to participate across age groups. Contrary to prior research (30) age emerged as a significant main effect on willingness to participate, with participants aged 35 or older reporting lower willingness compared to younger participants. While Cumulative Link Model likewise indicated a statistically significant effect, its bootstrap confidence intervals include zero, suggesting that the main effect of age may not be robust. Additionally, while burnout symptoms alone were not related to willingness to participate, age moderated this effect, indicating that older participants with higher burnout symptoms showed lower willingness. Bootstrap confidence intervals including zero suggest caution in interpreting these findings, which is supported by non-significant Cumulative Link Model results. Notably, neither of these effects were moderated by work experience.

### 4.2 Interpretations

The discrepancy between the high stated willingness to participate and the challenges encountered during recruitment for the NETfacts pilot study indicates that expressed intentions may not translate into actual engagement, pointing to additional, unaddressed barriers at the implementation level. As the reasons for a (lack of) willingness were not directly assessed in this study, the following interpretations are tentative and should be explored in further studies. Potential barriers for participation may reflect factors discussed in previous studies, such as (internalized) stigma and fear of adverse career consequences (28, 32) when disclosing own incident-related stress. Having experienced existing programs offered by one’s aid organization as insufficiently useful may further negatively influence the trust in new programs and contribute to recruitment difficulties. Our study further suggests that critical incident-related stress, burnout symptoms and age influences EMS’ participation willingness. Critical incident-related stress appears to lower participation barriers, indicating that EMS recognize event-related strain and response with openness to participate in interventions. However, this relationship could not be found for burnout. The average burnout score around the mid-point may indicate higher resilience or a genuinely lower level of burden among the participants. At the same time, the findings may also be interpreted in light of research suggesting that individuals with higher level of distress are more likely to avoid disclosing their perceived burden (50). However, burnout symptoms among EMS of age 35 and older and older age are associated with participation barriers. While age effects should be interpreted with caution due to indication of non-significance in Cumulative Link Models, prior research suggests higher internalized stigma and lower mental health literacy among older people (54). If these factors also had an effect on our results among EMS of age 35 and older remains to be investigated. Interestingly, our findings suggest no effect of work experience. However, the limited definition and operationalization of ‘work experience’ across different EMS roles (e.g., paramedics, emergency doctors) suggest caution in interpreting these findings, as responsibility and occupational tasks during the EMS-CII events vary across functions.

### 4.3 Implications

Despite sufficient expected usefulness and willingness to participate in NETfacts, the observed gap between intention and actual engagement, highlights the need for implementation strategies that align closely with participant preferences. Participants’ mixed preferences for group characteristics suggest the value of offering various, flexible participation options, including both online and in-person formats, and mixed groups with familiar EMS. This pattern suggests a trade-off between accessibility and relational depth: while low-threshold online formats may reduce practical and psychological barriers to participation, in-person settings may better support trust-building and interpersonal exchange. Furthermore, the age-sensitive main effect and its decoupling between burnout symptoms and willingness to participate highlights the need for targeted strategies, suggesting that the framing of such interventions may be important (e.g., foster team cohesion, or improve functionality). Awareness and recruitment efforts should normalize intervention engagement and emphasize the link between critical incident-related stress and mental health. Implementation can be supported by peers, influential role models, or initially providing opportunities to process personal critical experiences with a mental health professional, along with low-threshold participation options that enable gradual, accessible involvement. Importantly, interventions addressing critical incident-related stress appear to remain a key entry point and fostering intrinsic motivation to participate in such interventions essential for efficient recruitment.

### 4.4 Limitations

First, the expected usefulness and willingness to participate in NETfacts were each assessed with a single ordinal scaled item. This leaves room for interpretation of participants’ understanding of these concepts and their personal explanations for expected limited usefulness and willingness. Importantly, these constructs are inherently multidimensional (e.g., individual relevance, experienced burden, practically usefulness), indicating that single items may not be sufficient to fully capture them. Moreover, Cumulative Link Models were additionally conducted as sensitivity analyses, the results of moderation analyses with ordinal variables should still be interpreted with caution. Second, the definition of ‘work experience’ as years of service may restrain the validity of results for work experience, as different EMS functions have different responsibilities and occupational tasks and therefore different work experiences during EMS-CII events, influencing both critical incident-related stress and burnout symptoms. Third, burnout symptoms were assessed using the ProQOL, in which burnout is operationalized as compassion fatigue encompassing feelings of helplessness and difficulties in daily work life. Given the ongoing inconsistencies in burnout definitions (55), this operationalization may not fully capture the construct and leave characteristics such as depersonalization or emotional exhaustion out of consideration. Compared with other studies reporting higher burnout levels (3, 12), the present sample exhibited comparatively low symptom levels (51), which may be influenced both by the measurement approach or sample-specific characteristics. Fourth, we did not assess clinically relevant symptoms of posttraumatic stress, anxiety or depression, which have been identified as relevant mental health issues among EMS (1, 2, 4, 13). Therefore, this study may not fully represent the burden among EMS and its association with the expected usefulness and willingness to participate in NETfacts. Fifth, our sample may be subject to selection bias, as voluntary participation may be associated with generally higher openness toward mental health issues and greater willingness to participate in intervention programs. Therefore, the sample may not be representative of the EMS population in Germany.

## 5 Conclusion

EMS represents a high-risk group due to highly stressful work environments, with limited uptake of existing prevention programs. Our study highlights a gap between EMS’ willingness to participate and their actual participation rates. Age-sensitive recruitment strategies and intervention characteristics tailored to EMS’ preferences that address practical and psychological barriers are essential to enhance motivation to participate. Facilitating the access to early, low-threshold trauma-informed interventions such as NETfacts is an essential first step to implement effective interventions and mitigate the adverse effects of critical incidents among EMS.

## Data Availability

The raw data supporting the conclusions of this article will be made available by the authors, without undue reservation.
