# Supplementary material for: RESCUE- expected usefulness and willingness to participate in a trauma-informed group intervention for coping with traumatic work experiences in the emergency medical services
Source: Front Psychiatry. 2026 May 12;17:1846003. doi: 10.3389/fpsyt.2026.1846003 (PMC13202379; doi:10.3389/fpsyt.2026.1846003)
Supplement: Supplementary file 1 [file Table1.docx]

**Supplementary Material**

**Supplementary Table S1**

*Means, standard deviations, and spearman correlations with confidence intervals*

| Variable | *M* | *SD* | 1 | | 2 | 3 | 4 | 5 |
| --- | --- | --- | --- | --- | --- | --- | --- | --- |
|  |  |  | |  |  |  |  |  |
| 1. Critical incident-related stress (EMS-CII) | 23.72 | 19.20 | |  |  |  |  |  |
|  |  |  | |  |  |  |  |  |
| 2. Burnout symptoms (ProQOL) | 21.93 | 5.05 | | .24** |  |  |  |  |
|  |  |  | | [0.12, 0.35] |  |  |  |  |
|  |  |  | |  |  |  |  |  |
| 3. Expected usefulness of NETfacts | 3.39 | 1.08 | | .09 | -.12 |  |  |  |
|  |  |  | | [-0.03, 0.21] | [-0.24, 0.01] |  |  |  |
|  |  |  | |  |  |  |  |  |
| 4. Willingness to participate in NETfacts | 3.76 | 1.03 | | .22** | -.08 | .58** |  |  |
|  |  |  | | [0.10, 0.33] | [-0.20, 0.04] | [0.49, 0.65] |  |  |
|  |  |  | |  |  |  |  |  |
| 5. Age  (coded as 1 for ≥35 years and 0 for <35 years) | ≥ 35: *n* = 105 (41.02%)  < 35: *n* = 151 (58.98%) | | | .11 | .01 | -.08 | -.12* |  |
|  |  |  |  | [-0.01, 0.23] | [-0.11, 0.13] | [-0.20, 0.04] | [-0.24, -0.00] |  |
|  |  |  | |  |  |  |  |  |
| 6. Work experience  (coded as 1 for > 5 years and 0 for $\leq$ 5 years) | > 5: *n* = 168 (65.62%) | | | .12* | -.03 | .01 | -.02 | .47** |
|  | $\leq$ 5: *n* = 88 (34.38%) | | | [0.00, 0.24] | [-0.16, 0.09] | [-0.11, 0.13] | [-0.14, 0.11] | [0.37, 0.56] |
|  |  |  | |  |  |  |  |  |

*Note.* *N = 256; M* and *SD* are used to represent mean and standard deviation, respectively. Values in square brackets indicate the 95% confidence interval for each correlation, * indicates *p* < .05, ** indicates *p* < .01.

**Supplementary Table S2**

*Regression analyses including work experience as a moderator*

| Outcome and Model fit | Predictors | *b (SE)* | 95%CI (Boot) | *β* | *p* |
| --- | --- | --- | --- | --- | --- |
|  |  |  |  |  |  |
| **Outcome: Expected usefulness** |  |  |  |  |  |
| Model fit:  *F* (3, 252) = .67, *p* = .57, *R²_adj_* = -.00 | Constant | 3.39 (.12) | [3.17, 3.60] |  | < .001 |
|  | Critical incident-related stress (EMS-CII) | 0.00 (.01) | [-0.01, 0.02] | .08 | .548 |
|  | Work experience (coded as 1 for > 5 years and 0 for $\leq$ 5 years) | 0.00 (.14) | [-0.27, 0.27] | .00 | .998 |
|  | Moderation (work experience x critical incident-related stress) | 0.00 (.01) | [-0.01, 0.02] | .01 | .931 |
|  |  |  |  |  |  |
| **Outcome: Expected usefulness** |  |  |  |  |  |
| Model fit:  *F* (3, 252) = 1.25, *p* = .293, *R²_adj_* = .00 | Constant | 3.38 (.12) | [3.16, 3.58] |  | < .001 |
|  | Burnout symptoms (ProQOL) | -0.01 (.02) | [-0.06, 0.03] | -.07 | .560 |
|  | Work experience (coded as 1 for > 5 years and 0 for $\leq$ 5 years) | 0.02 (.14) | [-0.25, 0.29] | .01 | .907 |
|  | Moderation (work experience x burnout symptoms) | -0.02 (.03) | [-0.07, 0.04] | -.06 | .608 |
|  |  |  |  |  |  |
| **Outcome: Willingness to participate** |  |  |  |  |  |
| Model fit:  *F* (3, 252) = 4.41, *p* < .01, *R²_adj_* = .04 | Constant | 3.82 (.11) | [3.61, 4.00] |  | < .001 |
|  | Critical incident-related stress (EMS-CII) | 0.01 (.01) | [-0.00, 0.02] | .18 | .169 |
|  | Work experience (coded as 1 for > 5 years and 0 for $\leq$ 5 years) | -0.09 (.13) | [-0.33, 0.17] | -.04 | .519 |
|  | Moderation (work experience x critical incident-related stress) | 0.00 (.01) | [-0.01, 0.02] | .05 | .682 |
|  |  |  |  |  |  |
| **Outcome: Willingness to participate** |  |  |  |  |  |
| Model fit:  *F* (3, 252) = .94, *p* = .423, *R²_adj_* = -.00 | Constant | 3.78 (.11) | [3.58, 3.98] |  | < .001 |
|  | Burnout symptoms (ProQOL) | 0.00 (.02) | [-0.04, 0.04] | .01 | .908 |
|  | Work experience (coded as 1 for > 5 years and 0 for $\leq$ 5 years) | -0.04 (.14) | [-0.29, 0.22] | -.02 | .788 |
|  | Moderation (work experience x burnout symptoms) | -0.03 (.03) | [-0.08, 0.03] | -.11 | .319 |
|  |  |  |  |  |  |

*N* = 256; work experience coded as 1 for > 5 years and 0 for $\leq$ 5 years*;* 95%CI = bootstrap-based confidence interval, with 10000 times (non-parametric) bootstrap intervals; critical incident-related stress (EMS-CII = EMS Critical Incident Inventory) and burnout symptoms (ProQOL = Professional Quality of Life) were centered prior to calculation.

**Supplementary Table S3**

*Cumulative Link Model* *including age as a moderator*

| Outcome and Model fit | Predictors | *b* *= log(Odds)* *(SE)* | 95%CI (Boot) | *z* | *β* | *p* |
| --- | --- | --- | --- | --- | --- | --- |
|  |  |  |  |  |  |  |
| **Outcome: Expected usefulness**  Model fit: *χ²*(3) = 4.53, *p = .*209*,* AIC = 750.49, BIC = 775.30, *McFadden´s R²* = .01 | Critical incident-related stress (EMS-CII) | 0.01 (0.01) | [-0.01, 0.03] | 0.84 | 0.14 | .402 |
|  | Age (coded as 1 for ≥35 years and 0 for <35 years) | -0.31 (0.23) | [-0.77, 0.17] | -1.36 | -0.31 | .176 |
|  | Moderation (age x critical incident-related stress) | 0.01 (0.01) | [-0.02, 0.03] | 0.52 | 0.12 | .603 |
|  |  |  |  |  |  |  |
|  |  |  |  |  |  |  |
| **Outcome: Expected usefulness**  Model fit: *χ²*(3) = 5.01, *p* = *.*171*,* AIC = 750.01, BIC = 774.83, *McFadden´s R²* = .01 | Burnout symptoms (ProQOL) | -0.03 (0.03) | [-0.09, 0.03] | -0.88 | -0.14 | .378 |
|  | Age (coded as 1 for ≥35 years and 0 for <35 years) | -0.29 (0.23) | [-0.74, 0.21] | -1.25 | -0.29 | .210 |
|  | Moderation (age x burnout symptoms) | -0.03 (0.05) | [-0.13, 0.08] | -0.65 | -0.15 | .516 |
|  |  |  |  |  |  |  |
|  |  |  |  |  |  |  |
| **Outcome: Willingness to participate**  Model fit: *χ²*(3) = 19.65, *p < .*001*,* AIC = 695.53, BIC = 720.35, *McFadden´s R²* = .03 | Critical incident-related stress (EMS-CII) | .  0.03 (0.01) | [0.01, 0.05] | 3.11 | 0.51 | .  .002 |
|  | Age (coded as 1 for ≥35 years and 0 for <35 years) | -0.46 (0.24) | [-0.95, 0.01] | -1.97 | -0.46 | .049 |
|  | Moderation (age x critical incident-related stress) | -0.00 (0.01) | [-0.03, 0.02] | -0.17 | -0.04 | .865 |
|  |  |  |  |  |  |  |
|  |  |  |  |  |  |  |
| **Outcome: Willingness to participate**  Model fit: *χ²*(3) = 6.69, *p = .*083*,* AIC = 708.50, BIC = 733.32, *McFadden´s R²* = .01 | Burnout symptoms (ProQOL) | 0.02 (0.03) | [-0.04, 0.08] | 0.65 | 0.10 | .515 |
|  | Age (coded as 1 for ≥35 years and 0 for <35 years) | -0.38 (0.23) | [-0.88, 0.09] | -1.65 | -0.38 | .099 |
|  | Moderation (age x burnout symptoms) | -0.08 (0.05) | [-0.18, 0.01] | -1.87 | -0.43 | .061 |
|  |  |  |  |  |  |  |

*N* = 256; age coded as 1 for ≥35 years and 0 for <35 years*;* 95%CI = bootstrap-based confidence interval, with 1000 times (non-parametric) bootstrap intervals; critical incident-related stress (EMS-CII = EMS Critical Incident Inventory) and burnout symptoms (ProQOL = Professional Quality of Life) were centered prior to calculation.

**Supplementary Table S4**

*Cumulative Link Model* *including work experience as a moderator*

| Outcome and Model fit | Predictors | *b = log(Odds)*  *(SE)* | 95%CI (Boot) | *z* | *β* | *p* |
| --- | --- | --- | --- | --- | --- | --- |
|  |  |  |  |  |  |  |
| **Outcome: Expected usefulness** |  |  |  |  |  |  |
| Model fit: *χ²*(3) = 2.45, *p = .*485*,* AIC = 752.57, BIC = 777.39, *McFadden´s R²* = .00 | Critical incident-related stress (EMS-CII) | 0.01 (0.01) | [-0.01, 0.03] | 0.64 | 0.15 | .520 |
|  | Work experience (coded as 1 for > 5 years and 0 for $\leq$ 5 years) | 0.00 (0.24) | [-0.46, 0.47] | 0.01 | 0.00 | .994 |
|  | Moderation (work experience x critical incident-related stress) | 0.00 (0.01) | [-0.02, 0.03] | 0.17 | 0.04 | .867 |
|  |  |  |  |  |  |  |
|  |  |  |  |  |  |  |
| **Outcome: Expected usefulness** |  |  |  |  |  |  |
| Model fit: *χ²*(3) = 3.50, *p = .321,* AIC = 751.52, BIC = 776.34, *McFadden´s R²* = .00 | Burnout symptoms (ProQOL) | -0.02 (0.04) | [-0.09, 0.05] | -0.48 | -0.10 | .634 |
|  | Work experience (coded as 1 for > 5 years and 0 for $\leq$ 5 years) | 0.02 (0.24) | [-0.44, 0.52] | 0.09 | 0.02 | .932 |
|  | Moderation (work experience x burnout symptoms) | -0.03 (0.05) | [-0.12, 0.07] | -0.66 | -0.16 | .513 |
|  |  |  |  |  |  |  |
|  |  |  |  |  |  |  |
| **Outcome: Willingness to participate** |  |  |  |  |  |  |
| Model fit: *χ²*(3) = 16.15, *p < .*001*,* AIC = 699.03, BIC = 723.84, *McFadden´s R²* = .02 | Critical incident-related stress (EMS-CII) | 0.02 (0.01) | [-0.00, 0.04] | 1.52 | 0.36 | .129 |
|  | Work experience (coded as 1 for > 5 years and 0 for $\leq$ 5 years) | -0.05 (0.24) | [-0.50, 0.46] | -0.19 | -0.05 | .853 |
|  | Moderation (work experience x critical incident-related stress) | 0.01 (0.01) | [-0.02, 0.04] | 0.60 | 0.16 | .551 |
|  |  |  |  |  |  |  |
|  |  |  |  |  |  |  |
| **Outcome: Willingness to participate** |  |  |  |  |  |  |
| *χ²*(3) = 1.59, *p = .*661*,* AIC = 713.58, BIC = 738.40, *McFadden´s R²* = .00 | Burnout symptoms (ProQOL) | 0.01 (0.04) | [-0.06, 0.09] | 0.37 | 0.07 | .714 |
|  | Work experience (coded as 1 for > 5 years and 0 for $\leq$ 5 years) | 0.04 (0.24) | [-0.41, 0.53] | 0.16 | 0.04 | .873 |
|  | Moderation (work experience x burnout symptoms) | -0.05 (0.05) | [-0.14, 0.05] | -0.97 | -0.24 | .331 |
|  |  |  |  |  |  |  |
|  |  |  |  |  |  |  |

*N* = 256; work experience coded as 1 for > 5 years and 0 for $\leq$ 5 years; 95%CI = bootstrap-based confidence interval, with 1000 times (non-parametric) bootstrap intervals; critical incident-related stress (EMS-CII = EMS Critical Incident Inventory) and burnout symptoms (ProQOL = Professional Quality of Life) were centered prior to calculation.
